# Evaluation of empirical antibiotic use in diabetic foot infections at a tertiary hospital in Vietnam: A retrospective study

**DOI:** 10.1097/MD.0000000000040597

**Published:** 2024-11-15

**Authors:** Nam Quang Tran, Trang Nguyen Doan Dang, Cam Thai Nguyet Vo, Thu Thi Anh Nguyen, Quoc Nguyen Bao Pham, Minh Duc Do

**Affiliations:** a Department of Endocrinology, Faculty of Medicine, University of Medicine and Pharmacy at Ho Chi Minh City, Ho Chi Minh City, Vietnam; b Department of Endocrinology, University Medical Center Ho Chi Minh City, Ho Chi Minh City, Vietnam; c Department of Clinical Pharmacy, Faculty of Pharmacy, University of Medicine and Pharmacy at Ho Chi Minh City, Ho Chi Minh City, Vietnam; d Department of Pharmacy, University Medical Center Ho Chi Minh City, Ho Chi Minh City, Vietnam; e Department of Neurology, University Medical Center Ho Chi Minh City, Ho Chi Minh City, Vietnam; f Center for Molecular Biomedicine, University of Medicine and Pharmacy at Ho Chi Minh City, Ho Chi Minh City, Vietnam.

**Keywords:** diabetic foot, empirical antibiotics, local guidelines

## Abstract

Empirical antibiotic prescription guidelines were developed at the University Medical Center Ho Chi Minh City in 2020, which included recommendations for the use of antibiotics to treat diabetic foot infections (DFIs). This study investigated the treatment outcomes when implementing empirical antibiotic guidelines. This retrospective study included 120 inpatients with DFIs at the Department of Endocrinology, University Medical Center Ho Chi Minh City. This study had 2 periods (before and after implementation of hospital antibiotic guidelines): Period 1 from July 2019 to June 2020 and Period 2 from July 2021 to June 2022, with 60 random patients in each period. Treatment outcomes were assessed as follows: improvement (defined as the absence of fever and a white blood cell count within the normal range) at 72 hours and 7 days; duration of hospitalization; and clinical status at hospital discharge. After implementing empirical antibiotic guidelines, a greater proportion of improvement in the first 7 days of hospitalization (75.0% vs 56.7%, *P* = .03), and a shorter median duration of hospitalization (12.5 days vs 15.0 days, *P* = .02) were observed in patients with DFIs. All the patients showed improvement at the time of hospital discharge. The study findings revealed the encouraging effects of implementing empirical antibiotic prescription guidelines for DFI treatment.

## 1. Introduction

Type 2 diabetes mellitus is a chronic complex disease with the contributive roles of both genetic and environmental factors.^[1–4]^ The healthcare burden of type 2 diabetes is significant due to its diverse complications.^[5]^ Diabetic foot infections (DFIs) are the leading cause of nontraumatic amputations and hospitalization in patients with type 2 diabetes.^[6–8]^ Lower extremity amputations in patients with DFI result in a financial burden and reduced quality of life.^[9–12]^ Mortality rate can increase within 5 years after amputation, ranging from 39% to 80%. Successful management of DFIs should include optimal glycemic control, effective revascularization, surgical debridement, and optimal antimicrobial therapy.^[13]^ Among these measures, the inappropriate use of empirical antimicrobials and failure to cover etiological agents can lead to unfavorable clinical outcomes, including delayed wound healing, a higher risk of recurrent infection, and the need for amputation.^[14]^ Numerous factors, including geographic location, sensitivity patterns, duration and severity of infection, and prevalence of drug-resistant pathogens in patients, have been found to influence the bacterial features in DFIs.^[15]^ Additionally, the amputation risks associated with long-term use of broad-spectrum antibiotics have been investigated in clinical settings. The inappropriate use of broad-spectrum antibiotics is likely to increase the adverse effects of antibiotics, risk of antibiotic resistance, and cost of treatment.^[16]^ Therefore, antibiotic management guidelines for DFIs must be established and regularly updated in local hospitals.

In 2020, the University Medical Center (UMC) Ho Chi Minh City issued the empirical antibiotic guidelines for DFI (antibiotic UMC guidelines). These guidelines were derived from the Infectious Diseases Society of America 2012, the International Working Group on the Diabetic Foot 2019, and local microbiological findings from our hospital. In antibiotic stewardship programs, guidelines should be adapted for clinical practice, particularly for DFIs involving complex combinations of wound treatments and bacterial pathogens. This study was conducted to evaluate antibiotic use to treat DFIs in hospitals according to the UMC guidelines, with the following objectives: to investigate the characteristics of pathogens in DFIs, and to evaluate the outcomes of DFIs following the hospital-based guidelines.

## 2. Patients and methods

This is a retrospective study. The patients with DFI were recruited in 2 periods. The first group consisted of 60 patients in Period 1 (from July 2019 to June 2020) before the antibiotic guidelines were issued, and the second group consisted of 60 patients in Period 2 (from July 2021 to June 2022) after the guidelines were issued. Patients with type 2 diabetes (≥18 years) who were hospitalized for DFI in the Department of Endocrinology at UMC were included in this study. The presence of DFI was confirmed by the clinical diagnosis documented in the medical records. The exclusion criteria were co-infections, necrotizing fasciitis, pregnancy or lactation, renal replacement therapy (kidney transplant, peritoneal dialysis, hemodialysis), immunocompromised medical conditions (e.g., human immunodeficiency virus infection, cancer), and inadequate information in the records.

The sample size was estimated based on the appropriateness of antibiotic choices according to hospital guidelines. According to Huỳnh et al (2021), only 30% of 33 patients with DFI at UMC were prescribed empirical antibiotics in accordance with the Infectious Diseases Society of America 2012 guidelines, whereas previous studies reported a range of 31.3% to 83%.^[17–20]^ Thus, we incorporated 2 estimated proportions of guideline adherence into the calculation of the sample size: 30% for Period 1 and 70% for Period 2. A total of 120 patients with DFI were recruited in the 2 study periods (60 patients with DFI in each period). The baseline characteristics of all the participants were collected including age, gender, body mass index (BMI), estimated glomerular filtration rate (eGFR) levels, HbA1c levels, comorbidities, prior hospitalization for current foot ulcer, surgical debridement, amputation, revascularization procedure, duration of DFI, marker inflammation, grade of infection and presence of osteomyelitis.

In this study, the culture results of diabetic foot wound fluid samples collected within the first 7 days of admission were analyzed because most patients (80.8%) underwent wound culture sampling during this timeframe. Furthermore, the possibility of missing pathogens can be minimized by examining multiple episodes during this period, and these samples should be representative of the pathogens on admission.

The pathogens in DFI were identified by BD Phoenix M50 (Becton Dickenson, USA) or Vitek 2 Compact (Bio-Mérieux, France) (identification and antimicrobial susceptibility testing); cephalosporin/clavulanate combination disks and broth microdilution (extended-spectrum beta-lactamase (ESBL) testing); and AmpC induction test combined with phenotypic resistance to cephalosporin (AmpC testing).

Treatment effectiveness was evaluated by fever and white blood cell count at 72 hours and 7 days, the clinical status at the time of hospital discharge, duration of hospitalization, and the rate of amputations. Minor amputation was referred to as the surgical removal of the toes, while major amputation involved the amputation of a limb above the ankle.

IBM SPSS Statistics 25 software (IBM Corp., Armonk) was used for statistical analyses. Group comparisons were conducted using the Chi-squared or Fisher exact test for categorical variables and the *t*-test or Mann–Whitney U test for continuous variables. Statistical significance was set at *P* < .05.

This study was approved by the Ethics Council for Medical Research of the University of Medicine and Pharmacy at Ho Chi Minh City (protocol number 592/HĐĐĐ - ĐHYD on November 11, 2021).

## 3. Results

### 3.1. Baseline characteristics

Among 120 patients, the mean age of patients was 65.9 ± 11.0 years, and 40.8% were female. The median hemoglobin A1c was 9.2%. Peripheral vascular and peripheral neuropathy diseases were found in 40% and 19.2% of the patients, respectively. One-fourth of the patients had coronary artery disease (26.7%) or chronic kidney disease (27.5%). More than half (56.7%) of the patients required surgical debridement. Approximately one-fourth of the patients (24.2%) underwent amputation during hospitalization, with minor amputations accounting for 93.1% of these cases. Besides, endovascular procedures were necessary in 19.2% of the patients. Regarding infection characteristics, most patients had moderate DFI (66.7%) and one-third had severe DFI (33.3%). In addition, osteomyelitis was observed in 23.3% of the patients (Table 1).

**Table 1:** Demographic and clinical characteristics of the study participants (n = 120 patients).

Characteristics	Total number of patients (n = 120)	Period 1 (n_1_ = 60)	Period 2 (n_2_ = 60)	*P*-value
Age (yr), mean ± SD	65.9 ± 11.0	68.2 ± 11.9	63.5 ± 9.5	**.02**
Gender, *n* (%)				
Male	71 (59.2)	37 (61.7)	34 (56.7)	.58
Female	49 (40.8)	23 (38.3)	26 (43.3)	
BMI (kg/m^2^), median (IQR)	24 (21–26)	23 (21–26)	24 (22–26)	.56
eGFR (mL/min/1.73 m^2^), mean ± SD	65.3 ± 26.9	63.8 ± 24.3	66.8 ± 29.4	.55
HbA1c (%), median (IQR)	9.2 (7.7–11.0)	8.4 (7.4–10.3)	9.3 (8.1–11.6)	.11
Comorbidities, *n* (%)				
Hypertension	84 (70.0)	45 (75.0)	39 (65.0)	.23
Peripheral vascular disease	48 (40.0)	29 (48.3)	19 (31.7)	.06
Chronic kidney disease	33 (27.5)	16 (26.7)	17 (28.3)	.84
Exogenous Cushing’s syndrome	33 (27.5)	15 (25.0)	18 (30.0)	.54
Coronary artery disease	32 (26.7)	14 (23.3)	18 (30.0)	.41
Anemia	29 (24.2)	11 (18.3)	18 (30.0)	.14
Peripheral neuropathy	23 (19.2)	11 (18.3)	12 (20.0)	.82
Prior hospitalization for current foot ulcer, *n* (%)	52 (43.3)	29 (48.3)	23 (38.3)	.27
Surgical debridement, *n* (%)	68 (56.7)	38 (63.3)	30 (50.0)	.14
Amputation, *n* (%)	29 (24.2)	12 (20.0)	17 (28.3)	.29
Minor amputation	27 (22.5)	11 (18.3)	16 (26.7)	
Major amputation	2 (1.7)	1 (1.7)	1 (1.7)	
Revascularization procedure, *n* (%)	23 (19.2)	13 (21.7)	10 (16.7)	.49
Duration of infections				
< 1 month	79 (67.5)	47 (79.7)	32 (53.2)	**.005**
≥ 1 month	38 (32.5)	12 (20.3)	26 (44.8)	
Fever (> 38 °C)	30 (25.0)	18 (30.0)	12 (20.0)	.21
WBC (Giga/L), mean ± SD	13.2 ± 5.4	12.8 ± 4.3	13.6 ± 6.3	.41
CRP (mg/L), median (IQR)	78.5 (20.5–156.7)	78 (22.0–158.7)	85 (12.5–154.2)	.95
PCT (ng/mL), median (IQR)	0.16 (0.06–0.37)	0.16 (0.08–0.33)	0.16 (0.06–0.42)	.86
Grade of infections (IWGDF 2019), *n (%*)				
Moderate (grade 3)	80 (66.7)	44 (73.3)	36 (60.0)	.12
Severe (grade 4)	40 (33.3)	16 (26.7)	24 (40.0)	
Osteomyelitis, *n* (%)	28 (23.3)	12 (20.0)	16 (26.7)	.39

BMI, body mass index; CRP, C-reactive protein; eGFR, estimated glomerular filtration rate; HbA1c, hemoglobin A1c; IQR, interquartile range; IWGDF, the International Working Group on the Diabetic Foot; PCT, procalcitonin; SD, standard deviation; WBC, white blood cells.

*P*-values in bold are statistically significant.

### 3.2. Bacteria characteristics

Each bacterial strain was counted only once for each patient, regardless of the frequency of its presence in the culture samples. Organisms isolated from 81 patients (67.5%) within 7 days of admission are presented in Table 2. The most frequently isolated microorganisms were *Staphylococcus aureus* (34.5%), followed by *Klebsiella pneumoniae* (23.5%), *Escherichia coli* (14.8%), *Pseudomonas aeruginosa* (13.6%), and *Proteus mirabilis* (11.1%). Enterobacteriaceae was the predominant isolated cause of DFIs in this study, accounting for 58% of the patients.

**Table 2:** Proportion of isolated pathogens and antibiotic-resistant pathogens within the first 7 days of hospitalization in 2 groups (n = 81 patients).

Isolated pathogens	Total number of patients (n = 81)	Period 1 (n_1_ = 39)	Period 2 (n_2_ = 42)
Gram-positive bacteria			
*Staphylococcus aureus*	28 (34.6)	12 (30.8)	16 (38.1)
*Enterococcus faecalis*	3 (3.7)	2 (5.1)	1 (2.4)
*Staphylococcus epidermidis*	2 (2.5)	2 (5.1)	−
*Streptococcus agalactiae*	2 (2.5)	1 (2.6)	1 (2.4)
*Enterococcus raffinosus*	1 (1.2)	−	1 (2.4)
*Staphylococcus haemolyticus*	1 (1.2)	1 (2.6)	−
*Staphylococcus lugdunensis*	1 (1.2)	−	1 (2.4)
*Streptococcus oralis*	1 (1.2)	1 (2.6)	−
*Streptococcus pyogenes*	1 (1.2)	1 (2.6)	−
Gram-negative bacteria			
*Klebsiella pneumoniae*	19 (23.5)	11 (28.2)	8 (19.0)
*Escherichia coli*	12 (14.8)	8 (20.5)	4 (9.5)
*Pseudomonas aeruginosa*	11 (13.6)	3 (7.7)	8 (19.0)
*Proteus mirabilis*	9 (11.1)	3 (7.7)	6 (14.3)
*Morganella morganii*	7 (8.6)	4 (10.3)	3 (7.1)
*Enterobacter cloacae*	7 (8.6)	3 (7.7)	4 (9.5)
*Citrobacter freundii*	4 (4.9)	2 (5.1)	2 (4.8)
*Acinetobacter baumannii*	2 (2.5)	−	2 (4.8)
*Serratia marcescens*	2 (2.5)	−	2 (4.8)
*Burkholderia pseudomallei*	1 (1.2)	−	1 (2.4)
*Proteus vulgaris*	1 (1.2)	−	1 (2.4)
*Klebsiella aerogenes*	1 (1.2)	1 (2.6)	−
*Pantoea agglomerans*	1 (1.2)	−	1 (2.4)
*Pseudomonas putida*	1 (1.2)	−	1 (2.4)
Anaerobic bacteria			
*Bacteroides fragilis*	1 (1.2)	1 (2.6)	−
Antibiotic-resistant pathogens			
Methicillin-resistant *Staphylococcus aureus* (MRSA)	21 (25.9)	8 (20.5)	13 (31.0)
ESBL-producing gram-negative bacteria	18 (22.2)	11 (28.2)	7 (16.7)
AmpC β-lactamase–producing gram-negative bacteria	27 (33.3)	8 (20.5)	19 (45.2)
Carbapenem-resistant gram-negative bacteria	10 (12.3)	1 (2.6)	9 (21.4)

The data is presented as frequency (percentage).

ESBL, extended-spectrum beta-lactamases.

In terms of multidrug-resistant pathogens, the 4 categories of antibiotic-resistant pathogens were methicillin-resistant *Staphylococcus aureus* (MRSA), ESBL-producing gram-negative bacteria, AmpC-producing gram-negative bacteria, and carbapenem-resistant gram-negative bacteria (resistant to imipenem or meropenem). The findings showed no significant differences in the number of MRSA isolates and ESBL-producing rods per patient between the 2 periods. However, during Period 2, a substantial increase was observed in AmpC-producing rods (45.2% vs 20.5% in Period 1, *P* = .02) and carbapenem-resistant rods (21.4% vs 2.6% in Period 1, *P* = .02) (Table 2).

### 3.3. Compliance with the empirical antibiotic recommendation

All the patients in this study were administered empirical antibiotics for DFIs. In Period 2, the percentage of appropriate antibiotic choices based on hospital guidelines was 86.7%.

In our study, the proportions of patients who were administered 1, 2, or 3 types of antibiotics were 21.7%, 75.8%, and 2.5%, respectively. Vancomycin was used as the empirical antibiotic in most cases (62.5%), followed by ceftriaxone (28.3%), meropenem (24.2%), cefoperazone/sulbactam (23.3%), linezolid (10.8%), and ertapenem (6.7%). Comparison of the characteristics of the empirical antibiotic patterns between the 2 groups showed no statistically significant differences in antibiotics, antibiotic classes, or empirical antibiotic therapy (Table 3).

**Table 3:** Comparison of the empirical antibiotic use between 2 groups (n = 120 patients).

Antibiotics	Total number of patients (n = 120)	Period 1 (n_1_ = 60)	Period 2 (n_2_ = 60)	*P*-value
Vancomycin	75 (62.5)	40 (66.7)	35 (58.3)	.35
Ceftriaxone	34 (28.3)	15 (25.0)	19 (31.7)	.42
Meropenem	29 (24.2)	13 (21.7)	16 (26.7)	.52
Cefoperazone/sulbactam	28 (23.3)	17 (28.3)	11 (18.3)	.20
Linezolid	13 (10.8)	3 (5.0)	10 (16.7)	**.04**
Ertapenem	8 (6.7)	5 (8.3)	3 (5.0)	.36
Imipenem	7 (5.8)	1 (1.7)	6 (10.0)	.06
Clindamycin	6 (5.0)	6 (10.0)	−	**.01**

The data is presented as frequency (percentage).

*P*-values in bold are statistically significant.

### 3.4. Effectiveness of treatment

Both groups showed comparable improvements in fever and reductions in white blood cell counts at 72 hours of hospitalization (*P* = .20), whereas Period 2 had a significantly higher percentage of patients with improvement 7 days after hospitalization (*P* = .03). The length of hospital stay was much shorter in Period 2 as compared to Period 1 (12.5 days vs 15 days, *P* = .02). All the patients showed improvement upon hospital discharge. Meanwhile, the proportion of amputations did not differ between the 2 groups (*P* = .29) (Table 4).

**Table 4:** The results of treatment between 2 groups (n = 120 patients).

Outcomes	Total number of patients (n = 120)	Period 1 (n_1_ = 60)	Period 2 (n_2_ = 60)	*P*-value
Improvement after 72 hr, *n* (%)	65 (54.2)	29 (48.3)	36 (60.0)	.20
Improvement after 7 days, *n* (%)	79 (65.8)	34 (56.7)	45 (75.0)	**.03**
Length of hospitalization (days), median (IQR)	14 (10–19.75)	15 (12–21)	12.5 (9–17.75)	**.02**
Amputation, *n* (%)	29 (24.2)	12 (20.0)	17 (28.3)	.29
Improvement at the time of discharge, *n* (%)	120 (100)	60 (100)	60 (100)	>.99

*P*-values in bold are statistically significant.

## 4. Discussion

All the patients in our study had advanced DFI (graded by International Working Group on the Diabetic Foot 2019 guidelines), with an osteomyelitis prevalence of 23.3%. The microbiological material, wound fluid or pus, was collected 7 days after hospitalization for 80.8% of patients. A retrospective study conducted by Leibovitch et al (2021) examined the microorganisms present in patients with DFI within the first 2 weeks of admission, and found that 71% of patients were tested.^[21]^

According to our findings, the treatment of DFIs has become increasingly difficult owing to the rise in drug-resistant bacteria, including MRSA (25.9%), ESBL-producing gram-negative bacteria (22.2%), and carbapenem-resistant bacteria (12.3%). Our results align with the findings of various Asian studies that have shown growing resistance in DFIs.^[22]^ The presence of fluoroquinolone-resistant bacteria in DFIs is commonly reported because of the frequent use of oral antibiotics.^[23–25]^ Furthermore, a high proportion of ESBL-producing bacteria was reported by Zubair et al (44.7%) and Saidel-Odes et al (51.7% to 71.8%).^[26,27]^ Regarding *P. aeruginosa* strains, the prevalence in this study (13.6%) was comparable to the range reported in Korea (7.8% to 19.4%).^[25]^ Antibiotic use is common in patients with diabetes for the treatment of chronic infections, which may contribute to the emergence of resistant bacteria. In addition, several conditions, such as osteomyelitis, severe wounds, poorly perfused wounds, polymicrobial wounds, local antibiotic resistance, prior hospitalizations, admissions to tertiary hospitals, and comorbidities (peripheral arterial disease, kidney disease, etc.), are also considered risk factors for multidrug resistance.

In our sample, the coherence of hospital-based guidelines for empirical antibiotic use in Period 2 was considerably high at 86.7%. As mentioned in the Methods section, several previous studies reported the proportions aligning empirical antibiotic guidelines, such as Chisman (31.1%), Pence (59.7%), and Hand (83%).^[18–20]^ Improper antibiotics can be administered by physicians unfamiliar with the IWGDF classification and DFI guidelines. Prescribing broad-spectrum antibiotics in DFIs may require antibiotic stewardship, thereby lowering the resistance burden. Published studies on antibiotic resistance in DFIs suggest developing empirical guidelines instead of frequently prescribing conventional antibiotics.^[18–20,22]^ At our hospital, nearly all patients with DFI have been documented for the grade of infection and risk of infection with MRSA, *P. aeruginosa*, multi-resistant gram-negative bacteria, and anaerobic bacteria. In addition, when patients with severe DFIs are admitted to our emergency department, a multidisciplinary team typically discusses the treatment plans, including antibiotic options. This approach could help improve physicians’ compliance with hospital guidelines.

The patients in Period 2 of this study showed improvement in short-term outcomes (absence of fever and normal white blood cell count) as well as reduced average hospital stay as compared to Period 1, although the amputation intervention did not differ between the groups. Li et al (2020) similarly reported improved outcomes in the group receiving antibiotic management, including higher rates of consistent microbiological results (96.8% vs 43.5%) and shorter fever duration (1 day vs 7.5 days).^[28]^ Foot infections are associated with prolonged hospitalization, leading to an increase in multidrug-resistant infections and higher treatment costs.^[29]^ According to Haug et al, the proper duration of intravenous antibiotics could potentially reduce the length of hospital stay for patients with DFI.^[30]^ However, the lack of association between amputation and antibiotic choices was documented by Li et al (2020) and Pratama et al (2022).^[28,31]^ The indication for amputation could depend on multiple factors (ulcer grade, vascular intervention, comorbidities, and the presence of multi-resistant bacteria). Although appropriate antibiotics are part of the treatment, the association with amputation remains uncertain.

A primary strength of our study is that we recorded wound fluid/pus samples within 7 days of hospital admission to analyze the bacterial characteristics of patients with DFI. Besides, it is noticeable that our results compared the effectiveness of antibiotic treatment before and after applying hospital guidelines

Our study was based on a survey of medical records; therefore, there were some limitations: the clinical effectiveness of wound healing was not evaluated; there were inadequate data on the clinical classification of ulcer severity compared with the grade of infection; thus, antibiotics could lead to inconsistencies in the guidelines; preceding antibiotic exposure is yet to be recorded entirely; and we did not specifically classify microbiological samples as wound fluid, purulent discharge, or deep tissue samples.

Further studies could investigate the larger sample sizes of DFI patients, including those in orthopedic settings, to confirm the effectiveness of adherence to hospital guidelines. The duration of intravenous antibiotic therapy in hospitals should be assessed for its significance in antibiotic stewardship and long-term DFI treatment outcomes, including clinical improvements, ulcer recurrence, and reinfection.

## 5. Conclusions

The study results have demonstrated the benefits of applying hospital guidelines for DFIs. In comparison, during the period when empirical antibiotic guidelines were applied, patients probably improved their outcomes within the initial 7 days of hospitalization and showed a reduction in the median length of hospital stay. In addition, we identified a significant proportion of antibiotic-resistant bacteria in the study sample, including MRSA, ESBL-producing gram-negative bacteria, AmpC-producing gram-negative bacteria, and carbapenem-resistant bacteria.

## Acknowledgments

The authors would like to express our gratitude to the team members of the Department of Endocrinology at the University Medical Center Ho Chi Minh City for their valuable assistance.

## Author contributions

**Conceptualization:** Nam Quang Tran, Trang Nguyen Doan Dang, Minh Duc Do.

**Data curation:** Nam Quang Tran, Trang Nguyen Doan Dang, Cam Thai Nguyet Vo, Thu Thi Anh Nguyen, Quoc Nguyen Bao Pham.

**Formal analysis:** Nam Quang Tran, Cam Thai Nguyet Vo, Quoc Nguyen Bao Pham.

**Investigation:** Nam Quang Tran, Trang Nguyen Doan Dang, Cam Thai Nguyet Vo, Thu Thi Anh Nguyen, Quoc Nguyen Bao Pham.

**Methodology:** Trang Nguyen Doan Dang, Minh Duc Do.

**Supervision:** Nam Quang Tran, Trang Nguyen Doan Dang.

**Validation:** Thu Thi Anh Nguyen, Minh Duc Do.

**Visualization:** Minh Duc Do.

**Writing – original draft:** Nam Quang Tran, Trang Nguyen Doan Dang, Cam Thai Nguyet Vo, Minh Duc Do.

**Writing – review & editing:** Cam Thai Nguyet Vo, Minh Duc Do.
